# An attenuated coxsackievirus B5 mutant carrying VP1-N157K retains oncolytic potency against non-small cell lung cancer

**DOI:** 10.1016/j.omton.2025.200999

**Published:** 2025-05-23

**Authors:** Lifang Song, Bopei Cui, Qiushuang Gao, Chaoying Hu, Qian wang, Jialu Zhang, Yajing Li, Guanxing Liu, Yulong Fu, Ying Wang, Kelei Li, Xiaotian Hao, Fan Gao, Xing Wu, Qunying Mao, Zhenglun Liang, Yongxin Yu

**Affiliations:** 1 Division of Hepatitis and Enterovirus Vaccines, National Institutes for Food and Drug Control, NHC Key Laboratory of Research on Quality and Standardization of Biotech Products, NMPA Key Laboratory for Quality Research and Evaluation of Biological Products, Research Units of Innovative Vaccine Quality Evaluation and Standardization, Chinese Academy of Medical Sciences, Beijing 102629, China; 2 National Engineering Technology Research Center for Combined Vaccines, Wuhan 430207, China; 3 College of Integrative Medicine, Hebei University of Chinese Medicine, Hebei 050200, China; 4 Institute of Health and Medicine, Hefei Comprehensive National Science Center, Hefei 230601, China; 5 National Engineering Laboratory for AIDS Vaccine, School of Life Sciences, Jilin University, Changchun 130012, China; 6 Shanghai Institute of Biological Products Co., Ltd, Shanghai 201209, China; 7 Changchun Institute of Biological Products Co., Ltd, Changchun 130012, China; 8 School of Life Science and Biopharmaceutics, Shenyang Pharmaceutical University, Shenyang 110016, China; 9 Beijing Minhai Biotechnology Co., Ltd, Beijing 102600, China

**Keywords:** MT: Regular Issue, oncolytic virus, reverse genetics, virulence, safety, coxsackievirus, CAR-T, mRNA, lung cancer, intravenous injection, subcutaneous tumor model

## Abstract

Oncolytic virus therapy is a rapidly developing cancer treatment method. The development of oncolytic viruses often involves genetic modifications, such as increased expression of foreign proteins for enhancing the antitumor capabilities and knocking out of virulence loci for improving the safety. The wild-type coxsackievirus B5 (CV-B5/F) exhibits potent antitumor activity against non-small cell lung cancer. However, CV-B5 poses pathogenic risks to infants and young children, warranting further virulence attenuation to develop a safe strain. No attenuating locus has been reported for CV-B5. In this study, we attenuated the original strain CV-B5/F by low-temperature passage for 30 generations and identified the virulence locus N157K in the structural protein VP1 using reverse genetics analysis. The attenuated strain carrying VP1-N157K retained the antitumor capabilities as the original strain. In addition, an exploration of the potential attenuation mechanisms revealed that the VP1-N157K mutation site weakened the replication ability of CV-B5. In summary, we identified a key virulence locus of CV-B5 and constructed an attenuated strain, which retains the oncolytic activity of the wild-type strain against non-small cell lung cancer with increased safety.

## Graphical abstract

**Figure:**
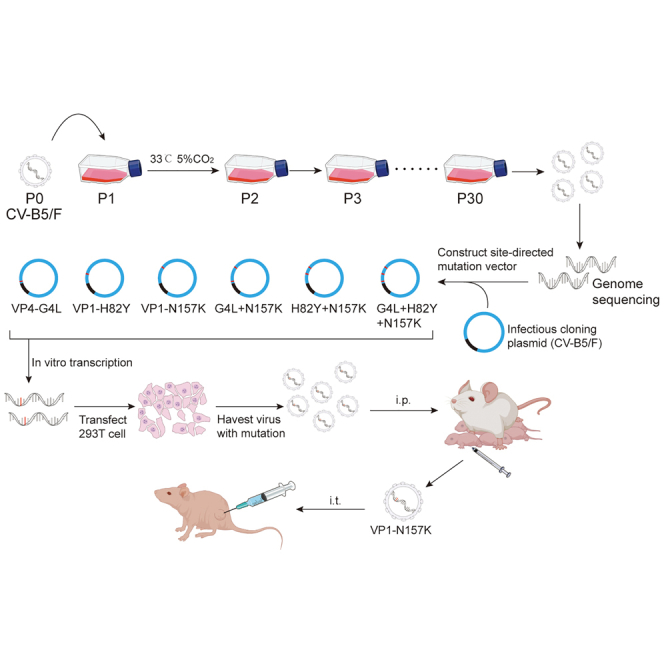

## Teaser

Lifang Song and colleagues identified a key virulence locus (VP1-N157K) in coxsackievirus B5 (CV-B5) through low-temperature passage and reverse genetics. The attenuated strain retains potent antitumor activity against non-small cell lung cancer while reducing pathogenic risks to infants. This study advances the development of safer oncolytic virus therapies.

## Introduction

Oncolytic viruses (OVs) are a class of naturally occurring or genetically engineered viruses that possess the ability to specifically recognize, infect, and lyse cancer cells and promote the immune system of the body to produce anticancer effects.^1^^,^^2^ Currently, four OVs are available on the market. These include Rigvir, which is approved in Latvia for the treatment of melanoma^3^; H101, approved in China for the treatment of head and neck cancer^4^; and Delytact, approved in Japan for the treatment of glioblastoma.^5^ Only talimogene laherparepvec (T-VEC) has been approved by the Food and Drug Administration and later by the European Commission for the treatment of metastatic melanoma.^6^ The modification of OVs involves several major aspects, including attenuation of the virus through modification of virulence sites^7^; genetic modification to enhance selective replication of the virus in tumor cells^8^; and increased expression of foreign proteins, such as cytokines, to enhance the immune response and achieve synergistic antitumor efficacy.^9^ Various enteroviruses have been reported to exhibit oncolytic effects, in addition to the already marketed Echovirus 7. For example, coxsackievirus A21 (CA21) exerts oncolytic effects in mouse models of malignant melanoma,^10^^,^^11^ bladder cancer,^12^^,^^13^ multiple myeloma,^14^ prostate cancer,^15^ and human breast tumors.^16^ Among these, the research on malignant melanoma has completed phase 2 clinical trials (NCT01636882), while the studies on breast cancer and bladder cancer have completed phase 1 clinical trials (NCT02316171). CV-B3 plays a positive role in the treatment of mouse models of lung,^17^^,^^18^^,^^19^ colon,^20^ endometrial,^21^ and breast cancers.^22^ In a previous research, we found that CV-B5 exhibits a definite antitumor effect on mouse models of non-small cell lung cancer.^23^ However, the direct use of the wild-type strains for *in vivo* injection poses safety issues; coxsackievirus has the potential to cause hand, foot, and mouth disease or severe neurological complications such as meningitis, warranting the need to obtain attenuated strains that retain the antitumor activity.^24^^,^^25^^,^^26^ Common attenuation strategies for enteroviruses include microRNA (miRNA)-mediated regulation^27^^,^^28^ and site mutation,^29^ to achieve attenuation and improve safety.

To the best of our knowledge, no clear reports of the attenuation sites in CV-B5 existed, and miRNA-mediated attenuation has not been attempted for this strain. In this study, we aimed to identify attenuating loci for CV-B5 to enable its use in OV therapy. We obtained a CV-B5 strain that exhibits both safety and antitumor capabilities by employing low-temperature passage and reverse genetics. Furthermore, we evaluated the key roles of the virulence sites. Our findings provided a foundation for basic research and further investigations on the antitumor effects of CV-B5.

## Results

### Low-temperature passage attenuated the virus with antitumor activity

**Figure 1:**
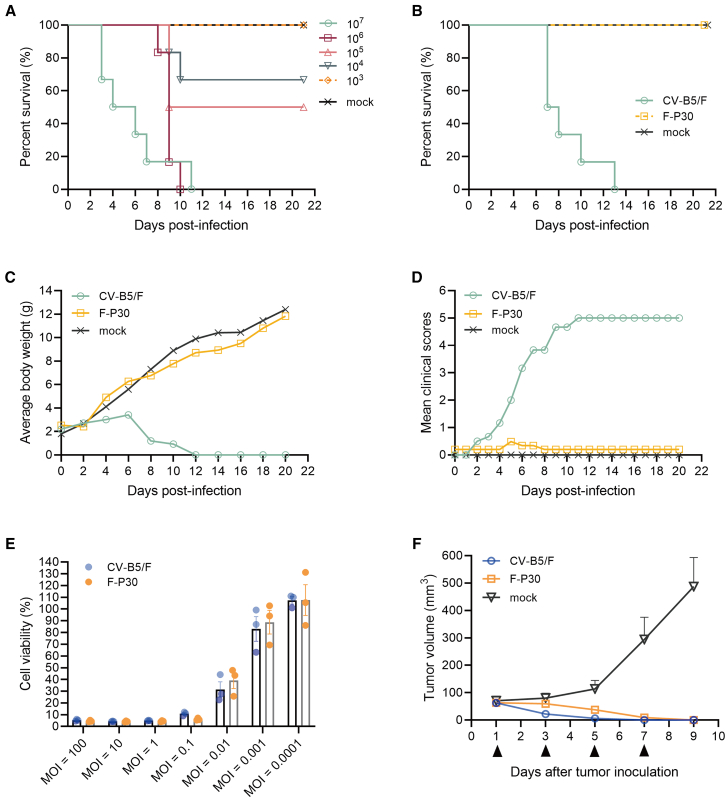
Virulence and antitumor ability of CV-B5/F and F-P30 (A) The LD_50_ of the CV-B5/F strain, determined by making a 10-fold gradient dilution and challenging 3-day-old suckling mice, intraperitoneally infected for a total of five gradients (*n* = 6). (B–D) Survival rate, mean clinical scores, and average body weights of suckling mice intraperitoneally infected with 35LD_50_ of CV-B5/F and F-P30 (mock groups were administered PBS) recorded up to 21 days postinfection (*n* = 6). (E) Viability of H1299 cells infected with CV-B5/F and F-P30 at an MOI of 0.0001–100 for 48 h (*n* = 3). (F) CV-B5/F and F-P30 were inoculated intratumorally with 1 × 10^7^ CCID_50_, and the changes in the volume of H1299 lung cancer tumors were observed (*n* = 5; mean ± SEM).

Adaptation of viruses to low temperatures is associated with reduced virulence in normal hosts.^30^ In this study, the CV-B5/Faulkner (CV-B5/F) strain was successively cold-passaged in Vero cells up to the 30th generation to obtain generation of strains, named F-P1 to F-P30. Viral titers were determined at 10-generation intervals. All the strains had titers in the range of 10^8.33^–10^8.75^ CCID_50_/mL (Table S1). For evaluating the changes in virulence of the passaged strains, suckling mice were intraperitoneally inoculated with virus diluents, and the lethal dose 50 (LD_50_) of CV-B5/F was determined. The survival of suckling mice showed a dose-dependent relationship with the viral titer, with an LD_50_ of 6.292 × 10^4^ CCID_50_ (Figure 1A). Furthermore, using 35LD_50_ (2.202 × 10^6^ CCID_50_) of CV-B5/F and F-P30 for suckling mice challenge, their survival rates, average body weight, and clinical scores were recorded for 21 days (Figures 1B–1D). All the suckling mice were killed by the parental wild type, CV-B5/F, at 13 days. Severe symptoms, such as body thinness and limb paralysis, were induced by the wild-type strain, and the average body weight was gradually decreased. In contrast, the cold-adapted virus, F-P30, did not cause death or induce clinical symptoms, and the average weight of mice gradually increased, indicating attenuation of the F-P30 virulence.

To investigate whether the cold-passaged virus strain retained its oncolytic activity, we tested the cytotoxicity of F-P30 on NCI-H1299 cells and its antitumor activity on NCI-H1299 cells transplanted into BALB/c nude mice. F-P30 was equally cytotoxic as CV-B5/F (both *p* > 0.05), with both the strains showing a dose-dependent effect (Figure 1E). The *in vivo* results showed that the F-P30 group exhibited complete tumor regression similar to the CV-B5/F group (Figure 1F) (both *p* > 0.05). This suggested that the *in vivo* and *ex vivo* antitumor activities of F-P30 in lung cancer were similar to those of CV-B5/F.

### Construction and verification of infectious clones with mutations in the low-passaged virulent strains

To identify mutations in the low-passaged strains, we sequenced the whole genome of CV-B5/F, F-P10, F-P20, and F-P30. A total of 7 nucleotide mutations were identified for low-temperature passaging up to F-P30 compared with the parental CV-B5/F, including three sites of meaningful mutations, all of which were stable starting F-P20. Among them, one nucleotide substitution site, VP4-A11T (VP4-G4L), and two reversal sites, VP1-C244T (VP1-H82Y) and VP1-C471A (VP1-N157K), were located in the structural protein (Table 1).

**Table 1:** Mutations in the low-temperature-passaged strain

No.	Region	nt/AA	Site	CV-B5/F	F-P1	F-P10	F-P20	F-P30	Mutation
1	VP4	nt	11	A	A	A	T	T	G4L
		AA	4	Gln	Gln	Gln	Leu	Leu	
2	VP3	nt	426	T	T	T	C	C	/
		AA	142	Pro	Pro	Pro	Pro	Pro	
3	VP1	nt	244	C	C	C	T	T	H82Y
		AA	82	His	His	His	Tyr	Tyr	
4		nt	471	C	C	C	A	A	N157K
		AA	157	Asn	Asn	Asn	Lys	Lys	
5	3C	nt	249	C	C	C	T	T	/
		AA	83	Phe	Phe	Phe	Phe	Phe	
6		nt	396	C	C	C	T	T	/
		AA	132	Thr	Thr	Thr	Thr	Thr	
7	3D	nt	234	T	T	T	T	C	/
		AA	78	Asp	Asp	Asp	Asp	Asp	

**Figure 2:**
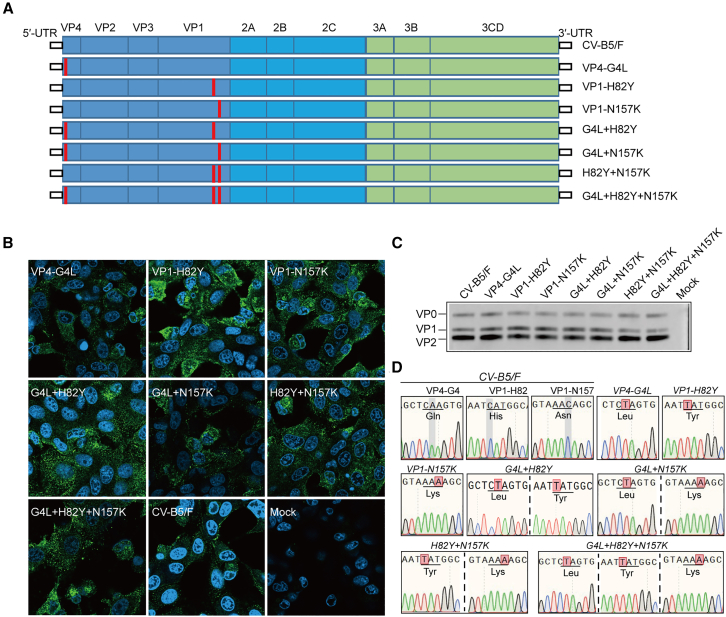
Construction and characterization of infectious clones of mutants (A) Schematic representation of CV-B5/F and mutations, including 5′-UTR, P1 (VP4, VP2, VP3, and VP1), P2 (2A, 2B, and 2C), P3 (3A, 3B, and 3CD), and 3′-UTR. (B and C) Vero cells were infected with CV-B5/F or mutants at an MOI of 0.01. Viral proteins were detected at 12 h postinfection using indirect immunofluorescence assay (B) and western blot analysis (C); scale bar, 10 μm. (D) Genome sequencing of CV-B5/F and mutants. The reddish shading indicates the mutation sites. Scale bars, 40 μm.

To further examine whether the attenuation of the CV-B5 cold-passaged virus strain was associated with mutations VP4-G4L, VP1-H82Y, and VP1-N157K, we used the infectious cDNA clone plasmid of CV-B/F constructed in a previous study^31^ and obtained single- and combined-site mutant strains using reverse genetics methods (Figure 2A). We verified each rescued virus strain, and, after infection of Vero cells, all the mutant strains exhibited cytopathic effects (CPE) similar to those of CV-B5/F (results not shown), and the CCID_50_ of all the mutant strains reached 10^8^ (Table S2). Moreover, the expression of CV-B5 proteins was detectable in the cytoplasm of all the mutant strains (Figure 2B), and western blot analysis revealed that all seven mutant strains expressed the structural proteins of CV-B5, namely VP0, VP1, and VP2 (Figure 2C). Simultaneouly, we verified the accuracy of the rescued viruses for each mutant strain via whole-virus genome sequencing (Figure 2D). This indicated the successful construction of the infectious clones of all mutant strains.

### Attenuation of the virulence of the VP1-N157K strain in suckling mice

**Figure 3:**
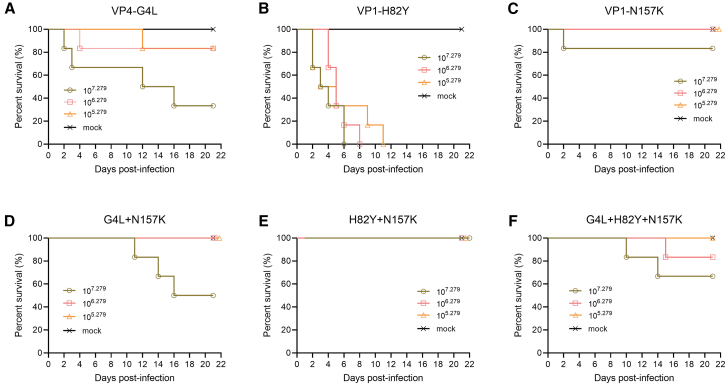
Survival rate of suckling mice for mutants Survival of 3-day-old BABL/c suckling mice challenged with the single-site mutant strains VP4-G4L (A), VP1-H82Y (B), and VP1-N157K (C) and the combined-site mutant strains G4L + N157K (D), H82Y + N157K (E), and G4L + H82Y + N157K (F), at doses of 10^7.279^ CCID_50_/mL (high dose), 10^6.279^ CCID50/mL (medium dose), and 10^5.279^ CCID_50_/mL (low dose), observed for 21 days (*n* = 6).

To investigate the virulence of the various mutant strains, the virus was administered to suckling mice at infective doses of 10^7.279^ (high dose), 10^6.279^ (medium dose), and 10^5.279^ (low dose) CCID_50_/mL, with continuous observation for 21 days, and survival, average body weight, and symptoms were recorded. The survival rate in the high-dose group of the VP4-G4L mutant strain was 33.3% (2/6), and the suckling mice grew slowly, whereas the survival rates in the medium- and low-dose groups were 83.3% (5/6), with some suckling mice exhibiting clinical symptoms (Figure 3A; Figure S1A). All the mice in the three VP1-H82Y mutant strain groups died (Figure 3B). In the high-dose group of the VP1-N157K mutant strain, only one suckling mouse died, the survival rate was 83.3% (5/6), and some mice showed mild symptoms between days 4 and 13, recovering by the day 14 and remaining well until day 21. The other two groups survived (Figure 3C; Figure S1C). Therefore, the mutation at the VP1-H82Y site had no attenuating effect, the VP4-G4L mutation had some effect, and the VP1-N157K strain has weaker virulence, compared to the above two strains.

To further explore whether the combination of the VP1-N157K mutation would affect the virulence of the VP4-G4L and VP1-H82Y mutant strains, suckling mice were challenged with the G4L + N157K, H82Y + N157K, and G4L + H82Y + N157K triple-mutant strains at the three aforementioned doses, with continuous observation for 21 days, and survival, weight, and symptoms were recorded. In the G4L + N157K mutant strain challenge groups, only mice in the high-dose group died, and the survival rate was 50% (3/6), whereas no deaths occurred in the other dose groups, indicating an increased survival rate compared with that in the VP4-G4L single-treatment group (Figures 3A and D). For the H82Y + N157K mutant strain, the survival rate was 100% (6/6) across all dose groups, whereas the survival rate for the VP1-H82Y single-mutation strain at all three challenge doses was 0% (0/6) (Figures 3B and 3E). The survival rates in the high- and medium-dose groups of the G4L + H82Y + N157K mutant strain were 66.7% (4/6) and 83.3% (5/6), respectively (Figure 3F). Thus, a combination with the VP1-N157K mutation effectively increased the survival rate of suckling mice challenged with high doses of the VP4-G4L and VP1-H82Y single-mutation strains, indicating that the VP1-N157K single-mutation site may be the cause of the reduced virulence of the F-P30 strain.

### Viral load and pathological changes

**Figure 4:**
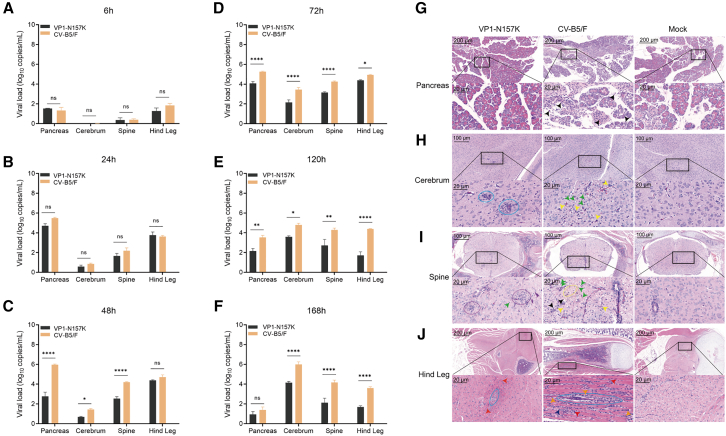
Viral loads and pathological changes in various tissues and organs of suckling mice (A–F) Viral loads in tissues and organs, such as the pancreas, brain, spine, and hind legs, of suckling mice at 6, 24, 48, 72, 120, and 168 h after intraperitoneal inoculation of VP1-N157K and CV-B5/F (*n* = 3). (G–J) Pathological changes in the pancreas (G), cerebrum (H), spine (I), and hind legs (J) after intraperitoneal inoculation of the attenuated strain VP1-N157K and the parental strain CV-B5/F at the same CCID_50_ for 5 days (*n* = 3), where sky blue ovals represent perivascular glial cell proliferation, purple circle represents glial nodular lesion, yellow arrows represent glial cell proliferation, yellow circle represents a loose matrix, green arrows indicate neuronal cell necrosis, black arrows represent lymphocytes; orange arrows represent tissue cell proliferation, and red arrows represents neutrophils. ∗*p* < 0.05; ∗∗*p* < 0.01; ∗∗∗∗*p* < 0.0001; ns, not significant.

To study the reason for the reduced virulence of the VP1-N157K strain in suckling mice, we intraperitoneally inoculated 3-day-old BALB/c suckling mice with CV-B5/F and the attenuated VP1-N157K strain at a titer of 10^7.279^ CCID_50_. Viral loads were determined in tissues with tropism, such as the pancreas, cerebrum, spinal cord, and hind leg muscles at 6 (Figure 4A), 24 (Figure 4B), 48 (Figure 4C), 72 (Figure 4D), 120 (Figure 4E), and 168 h (Figure 4F). Within 24 h, both the CV-B5/F and attenuated VP1-N157K strains were primarily present in the pancreas and hind leg muscles, with lower viral loads in the brain and spinal cord. Starting at 48 h, the viral loads in the brain and spinal cord gradually increased, and the CV-B5/F loads were significantly higher than those of the attenuated VP1-N157K strain (*p* < 0.05 for all comparisons). Therefore, after intraperitoneal injection, CV-B5 first infects the peripheral tissues, such as the pancreas, and then re-enters the bloodstream to reach neural tissues, such as the brain and spinal cord. At this time, the viral loads in the brain and spinal cord of mice challenged with the attenuated VP1-N157K strain were lower than those in mice challenged with CV-B5/F, which may be one of the reasons for the better survival of mice challenged with the attenuated VP1-N157K strain.

To further investigate the histopathological changes in the tissues of suckling mice challenged with the attenuated VP1-N157K strain, we inoculated 3-day-old BALB/c suckling mice with CV-B5/F and the attenuated VP1-N157K strain at a titer of 10^7.279^ CCID_50_. When the suckling mice in the CV-B5/F group were in a critical condition (around day 5 post-inoculation), we conducted histopathological studies on the pancreas, brain, spinal cord, and hind legs of mice from both groups at the same time points, with three blank control suckling mice. No significant pathological changes were observed in the pancreas of suckling mice challenged with the attenuated VP1-N157K strain, whereas CV-B5/F infection caused pancreatitis, with peri-acinar infiltration of inflammatory cells around the pancreatic acinar cells, primarily consisting of lymphocytic infiltration (black arrows), further indicating a reduced tropism of the attenuated VP1-N157K strain for the pancreas (Figure 4G). In the thalamic region, the VP1-N157K attenuated strain group mainly showed perivascular glial proliferation (within the sky blue box), with no other significant abnormalities observed, whereas the CV-B5/F-challenged group, in addition to glial cell proliferation (yellow), exhibited a large number of necrotic neurons, indicating more severe lesions (green) (Figure 4H). In the spinal cord, the VP1-N157K attenuated strain group mainly showed glial nodular lesion (within the purple circle), whereas the CV-B5/F-challenged group exhibited obvious necrosis of neuronal cells in the spinal cord (green arrows), accompanied by glial cells (yellow arrows), inflammatory cell infiltration (primarily lymphocytes, black arrows), and a loose matrix (within the yellow circle) (Figure 4I). This indicated that the pathological changes caused by CV-B5/F throughout the spinal cord were more severe than those caused by the attenuated VP1-N157K strain. In the hind leg tissues of suckling mice, the hind leg muscle fibers in the VP1-N157K attenuated strain group showed very mild eosinophilic degeneration (orange arrows) with tissue cell proliferation and muscle fiber regeneration and repair (within the sky blue circle), whereas the CV-B5/F-challenged group showed interstitial inflammatory cell infiltration in the hind leg muscles (including neutrophils and lymphocytes, red and black arrows) and tissue cell proliferation (orange arrows) with muscle fiber regeneration and repair (see multinucleated giant cell myotubes, within the sky blue circle) (Figure 4J). This further indicated that the pathological changes in the main tropic tissues and organs, such as the pancreas, brain, spinal cord, and hind legs, were significantly weaker in the attenuated VP1-N157K strain group than in the wild-type CV-B5/F group.

### Attenuation of replication ability may be the main reason for the reduced virulence of the VP1-N157K mutant strain

**Figure 5:**
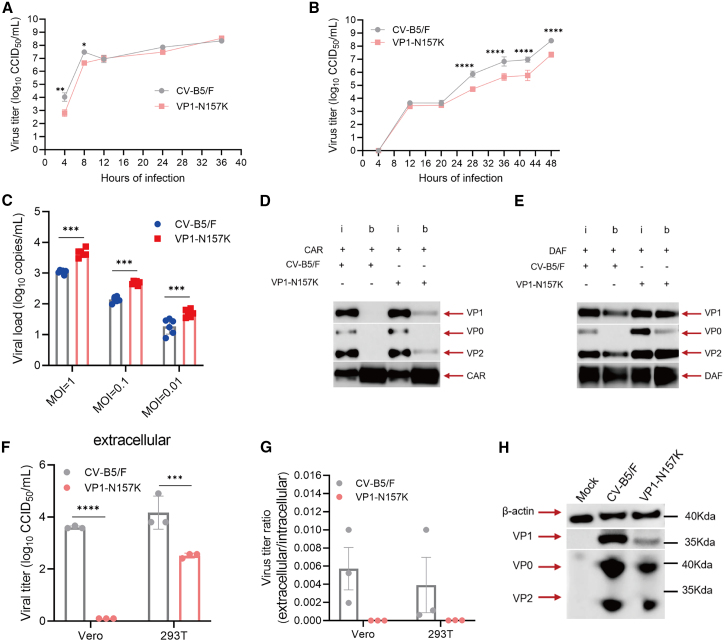
Investigation of the mechanism of attenuation of VP1-N157K (A and B) Growth kinetics of Vero cells infected with CV-B5/F or VP1-N157K at an MOI of 5 and 0.001 indicate that, at a higher MOI of 5, the growth capacity of VP1-N157K is weaker than that of the CV-B5/F wild-type strain in the early stage (A), and, at a low MOI of 0.001, the growth capacity of the VP1-N157K strain is significantly weaker than that of the CV-B5/F wild-type strain over time (B) (*n* = 3). (C) Viruses infect but do not enter cells at 4°C, and, after coincubation with Vero cells at 4°C for 3 h with different MOIs, unbound viruses were washed away, and qPCR was used to detect the number of viral copies attached to the cell surface (*n* = 3). (D and E) Detection of the binding capacity of the VP1-N157K and CV-B5/F strains to CAR and DAF receptors, respectively, using pull-down experiments; the binding strength of the VP1-N157K strain to CAR and DAF was not weaker than that of the parental strain. (F and G) The same concentration of VP1-N157K and CV-B5/F mRNA was transfected into 293T and Vero cells, and, after 8 h, the virus titer of the VP1-N157K attenuated strain was significantly lower than that of the CV-B5/F wild-type strain (F), and the extracellular/intracellular virus titer ratio of the CV-B5/F strain was higher than that of the VP1-N157K strain (G) (*n* = 3). (H) The same concentration of VP1-N157K and CV-B5/F mRNA was transfected into 293T cells, and, after 24 h, the protein expression of the VP1-N157K attenuated strain in 293T cells was lower than that of the CV-B5/F strain. ∗*p* < 0.05; ∗∗*p* < 0.01; ∗∗∗*p* < 0.001; ∗∗∗∗*p* < 0.0001.

To further verify the reasons for reduced virulence of the VP1-N157K mutation, we investigated the mechanism of attenuation. Virus infection of cells mainly involves the processes of entry, replication, and exit, which virulence research has focused on. First, we explored the growth kinetics of the CV-B5/F and VP1-N157K strains at a multiplicity of infection (MOI) of 5 or 0.001. At a high MOI of 5, the titer of CV-B5/F was higher than that of VP1-N157K from 4 to 8 h (*p* < 0.05), and, after 12 h, the titers of the two strains were consistent (Figure 5A). In contrast, at a low MOI of 0.001, the titers of both the strains remained low within 20 h, and, as time progressed, the growth capacity of the CV-B5/F strain was significantly higher than that of the VP1-N157K strain (Figure 5B) (*p* < 0.0001), indicating that the replication capacity of the CV-B5/F strain may be stronger than that of the VP1-N157K strain. When virus infects cells, binding without entry occurs under conditions of 4°C.^32^ We infected Vero cells with the CV-B5/F and VP1-N157K strains at 4°C for about 3 h at MOIs of 1, 0.1, and 0.01. The load of the VP1-N157K strain attached to the cell surface was higher than that of the CV-B5/F strain (Figure 5C) (*p* < 0.001). CV-B5 infects cells mainly by entering the cells via decay accelerating factor (DAF) and CAR. To explore the reason for the enhanced attachment ability of the VP1-N157K strain, we performed pull-down experiments and found that the binding ability of the VP1-N157K strain to CAR and DAF was similar to that of the CV-B5/F strain (Figures 5D and 5E). Similar phenomena were observed in the surface plasmon resonance (SPR) experiments (Figure S3). This suggested that the attenuation of VP1-N157K was not related to the receptor.

To further explore the differences in the replication capacity between the two strains, we transfected Vero and 293T cells with mRNA of the two strains at the same dose and found that the titer of the CV-B5/F strain in the supernatant of both the cell strains was higher than that of the VP1-N157K strain at 8 h post-transfection (Figure 5F) (*p* < 0.001), and the ratio of extracellular to intracellular titers of the CV-B5/F strain was higher than that of the VP1-N157K strain (Figure 5G). At 24 h post-transfection, the expression levels of the structural proteins of the CV-B5/F strain in 293T cells were higher than those in the VP1-N157K strain (Figure 5H). This indicated that the replication and release capabilities of the VP1-N157K strain in cells were significantly weaker than those of the CV-B5/F strain, which could explain the reduced virulence of the VP1-N157K strain.

### VP1-N157K attenuated strain retains tumor growth inhibitory properties similar to the parental strain

**Figure 6:**
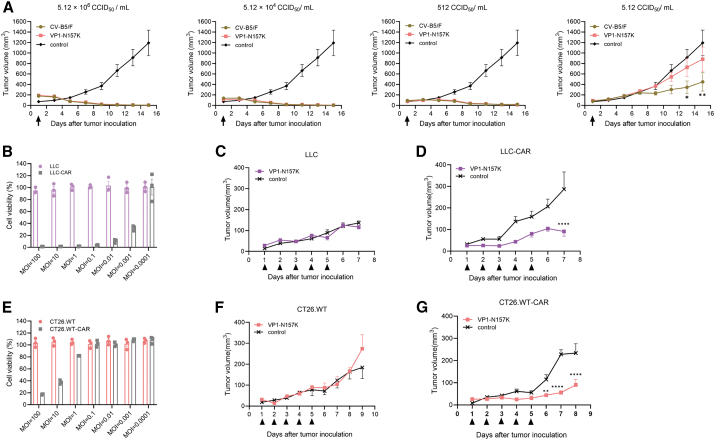
The oncolytic characteristics of the VP1-N157K attenuated strain (A) *In vivo* oncolytic evaluation of H1299 lung cancer by different titers of CV-B5/F and VP1-N157K strains, with a single intratumoral injection of 5.12 × 10^6^ CCID_50_/mL, 5.12 × 10^4^ CCID_50_/mL, and 512 CCID_50_/mL. Both the CV-B5/F and VP1-N157K strains were able to significantly reduce the tumor volume. When the virus titer was reduced to 5.12 CCID_50_/mL, the CV-B5/F strain effectively inhibited tumor growth, whereas the VP1-N157K strain showed a significant reduction in its ability to inhibit the increase in tumor volume (*n* = 5). (B–D) *In vitro* and *in vivo* killing of murine lung cancer LLC and LLC-CAR by the VP1-N157K attenuated strain; no effect on *in vitro* killing and *in vivo* oncolysis of LLC was observed (B and C), whereas overexpression of CAR resulted in effective delay in tumor growth (D) (*n* = 5). (E–G) *In vitro* and *in vivo* killing of murine colon cancer CT26.WT and CT26.WT-CAR by the VP1-N157K attenuated strain; no effect on *in vitro* killing and *in vivo* oncolysis of CT26.WT was observed (E and F), whereas overexpression of CAR resulted in effective delay in tumor growth (G) (*n* = 5). ∗*p* < 0.05; ∗∗*p* < 0.01; ∗∗∗∗*p* < 0.0001.

To further investigate the oncolytic effects of the VP1-N157K strain after its replication capacity was weakened, we utilized a lung cancer BALB/c nude mouse model previously established in our laboratory using NCI-H1299 lung cancer cells to study the oncolytic ability of the attenuated VP1-N157K strain. We evaluated the oncolytic effects of the CV-B5/F and VP1-N157K strains *in vivo* after a 100-fold gradient dilution with a titer of 5.12 × 10^6^ CCID_50_/mL. When the virus titer was between 512 and 5.12 × 10^6^ CCID_50_/mL, a single dose of the attenuated strain, VP1-N157K, showed oncolytic effects similar to those of the wild-type strain CV-B5/F (*p* > 0.05), both of which significantly reduced the tumor volume (Figure 6A). However, when the virus titer was reduced to 5.12 CCID_50_/mL, the wild-type strain still effectively inhibited tumor growth, but the attenuated strain showed a significantly reduced ability to inhibit tumor growth (Figure 6A). This indicated that the CV-B5/F strain is more potent than the VP1-N157K strain, although the maximum effect is the same.

Our previous experiments also confirmed that CV-B5/F’s selective infection of cancer cells depended on the cell surface receptor CAR. Murine lung cancer cells, LLC, and murine colon cancer cells, CT26.WT, express very low levels of CAR on their surfaces; hence, CV-B5/F rarely enters LLC and CT26.WT cells, resulting in poor killing efficacy against these two cell lines. However, after overexpressing CAR, the parental CV-B5/F strain exhibited enhanced *in vitro* cytotoxicity against LLC-CAR and CT26.WT-CAR cells and also demonstrated increased oncolytic activity in tumor-bearing mice models constructed from these two types of cells.^23^ To investigate whether the cytotoxic and oncolytic capacities of the attenuated VP1-N157K strain are consistent with those of the parental strain, we investigated the infection capability of the VP1-N157K attenuated strain with two cell lines stably transfected with the CAR receptor, LLC-CAR, and CT26.WT-CAR. The VP1-N157K strain exhibited a strong cytotoxic effect on both the cell lines that stably expressed CAR (Figure 6B and 6E), indicating the dependence of the VP1-N157K strain on the CAR receptor, which is consistent with that of the parental strain. Further experiments were conducted using the previously established tumor-bearing mouse models of LLC, LLC-CAR, CT26.WT, and CT26.WT-CAR to verify the relationship between the *in vivo* oncolytic effects mediated by the VP1-N157K strain and the expression of CAR receptors. The VP1-N157K strain had no oncolytic effect on LLC and CT26.WT tumor-bearing mice (Figures 6C and F). After stable transfection with CAR, the VP1-N157K strain could delay tumor growth in LLC-CAR and CT26.WT-CAR tumor-bearing mice, with a significant difference compared to the untreated group (*p* < 0.01) (Figures 6D and G). This proved that the entry mechanism of the VP1-N157K strain was the same as that of the parental strain, both of which were related to the expression of receptors on the cell surface. Under conditions of high CAR expression, the entry capability of the VP1-N157K strain was enhanced, leading to better oncolytic effects.

## Discussion

CV-B5 belongs to group B of enteroviruses and often causes aseptic meningitis, brainstem encephalitis, and other conditions in infants and young children.^33^^,^^34^ In our previous studies, we found that CV-B5/F has a good oncolytic effect on the BALB/c nude mouse model of H1299 non-small cell lung cancer, both *in vitro* and *in vivo*.^23^ However, CV-B5 is still virulent and poses safety risks when used clinically, requiring further attenuation. In this study, we passaged the original strain CV-B5/F at low temperatures until the 30th generation and found three mutation sites, namely VP4-G4L, VP1-H80Y, and VP1-N157K. Among them, the VP1-N157K mutation showed a more pronounced attenuation effect in the suckling mouse challenge model and still showed good antitumor ability against the BALB/c nude mouse model of non-small cell lung cancer.

OVs offer a promising cancer treatment method; however, as live viruses, their safety has attracted widespread attention. Enhancing the safety of OVs is a prerequisite for their widespread application, and common methods include genetic and targeted modifications, such as deletion of pathogenic genes, insertion of foreign genes, and mutations in virulence sites. The marketed oncolytic herpes simplex virus T-VEC is a safe strain obtained by knocking out the virulence site ICP34.5.^35^^,^^36^ For CV-B3, a virus with good oncolytic ability, safety concern is the biggest obstacle in its application. Researchers often construct different miRNAs targeting the viral genome and continuously optimize the sequence to improve stability and safety, while retaining the oncolytic characteristics of CV-B3, reducing its toxic effects on normal tissues.^37^^,^^38^ Research on CV-B5 has been scarce, and the only studies that have been conducted have shown that the 95th amino acid of the capsid protein VP1 is located on the outer surface of the viral particle, which may be involved in the interaction between the virus and cells and increased viral infection^39^^,^^40^; no other virulence sites have been unambiguously reported. Therefore, obtaining an attenuated virus through direct modification is difficult. Low-temperature passage is a common method to obtain a weak virulent strain.^41^ Therefore, in this study, we used low-temperature passage to obtain an attenuated strain with reduced virulence and retained oncolytic ability and analyzed the mutation sites via second-generation sequencing. Site-directed mutagenesis was used to study whether it is an attenuation site, obtaining a mutant strain VP1-N157K with reduced virulence that retained most, but not all, of the oncolytic ability of the parental strain.

The interaction between viruses and cell surface receptors is a key step in mediating viral infections.^42^^,^^43^ Non-enveloped single-stranded RNA viruses, such as CV-B3 and CV-B5, mainly enter host cells through the primary receptor, CAR, and the auxiliary receptor, DAF.^44^ Changes in the puff region of VP2 and the knob region of VP3 in the structural proteins of CV-B3 may affect the binding of the virus to DAF, and mutations can affect the virus entry.^45^^,^^46^^,^^47^ As the VP1-N157K site is located on VP1, it is speculated that it affects its ability to bind to receptors. We used mouse lung cancer cells LLC and mouse colon cancer cells CT26WT with low CAR expression. After stable CAR transfection, we found that the *in vivo* and *in vitro* killing ability of the virus in stable cell lines was enhanced. Simultaneously, by studying the ability of VP0, VP1, and VP2 to interact with CAR and DAF, we found that the binding ability of the mutant strain was not reduced. These results suggest that the VP1-N157K mutation does not affect the viral infection of host cells.

The regulation of interferon expression is crucial for virus replication.^48^ Studies have shown that the VP1 E83K mutation in the foot-and-mouth disease virus can promote virus replication by targeting mitochondrial antiviral signaling protein (MAVS) to inhibit the type-I interferon response.^49^ However, this study using interferon-deficient cells found a difference in replication, suggesting the existence of other mechanisms independent of interferons. The CV-B3 virus with VP2-Y254F or VP2-Y254F + VP2-Y240F mutations has an attenuating effect mainly because the cleavage of VP0 into VP2 and VP4 is defective, which affects the formation of viral assembly intermediates, weakens the growth ability of the virus, and ultimately reduces pathogenicity,^50^ which is a limitation. Whether the reduced replication ability of the VP1-N157K strain is also related to defects in viral assembly requires further research. Although the VP1-N157K strain has attenuated virulence, it still elicits certain inflammatory responses in the tissues and organs of suckling mice. Additionally, it has not been validated for safety in non-human primates. Therefore, it would not probably be sufficient for clinical applications. Although the VP1-N157K attenuated strain retains oncolytic properties similar to those of the parental strain, a higher concentration range of the virus is required to achieve the desired effect. In addition, the CV-B5 OV only demonstrated relatively good antitumor efficacy in the H1299 lung cancer mouse model, while its oncolytic effects were relatively poor in other lung cancer models such as A549 and H460 (Figure S4). This indicates the necessity to employ new strategies to enhance the oncolytic potency of CV-B5.

In summary, we obtained an attenuated CV-B5 strain with reduced virulence that retained its oncolytic ability through low-temperature passage and verified the attenuation site VP1-N157K using reverse genetics. This reduction in virulence was mainly related to the weakened replication ability of the virus, and the VP1-N157K attenuated strain exhibited the same oncolytic characteristics as the original strain. This study provides ideas for attenuation research on CV-B5 and offers safety assurance for the use of CV-B5 in antitumor therapy.

## Materials and methods

### Cells

African green monkey kidney cells (Vero) were cultured in minimum essential medium (MEM); mouse colon carcinoma cells (CT26.WT), CT26.WT cells with stably expressing coxsackievirus and adenovirus receptor (CAR) (CT26WT-CAR), and human non-small cell lung cancer cells (NCI-H1299) were cultured in RPMI 1640; human embryonic kidney cells 293T, mouse Lewis lung cancer (LLC) cells, and LLC cells with stable CAR transfection were cultured in Dulbecco's Modified Eagle Medium (DMEM). All cell culture media contained 10% fetal bovine serum (FBS) and 1% penicillin-streptomycin mixture. All the cells were cultured at 37°C in a 5% CO_2_ atmosphere. All the cells were purchased from ATCC and stored in the Division of Hepatitis and Enterovirus Vaccines of National Institute for Food and Drug Control (NIFDC), China.

### Viruses

Coxsackievirus B group 5 (CV-B5/F, GenBank: AF114383) was purchased from ATCC and was stored in the Division of Hepatitis and Enterovirus Vaccines of NIFDC. F-P10, F-P20, and F-P30 were obtained by passage of CV-B5/F at 33°C in an atmosphere of 5% CO_2_. The VP4-G4L, VP1-H82Y, VP1-N157K, VP4-G4L + VP1-N157K, VP1-H82Y + VP1-N157K, and VP4-G4L + VP1-H82Y + VP1-N157K strains were obtained through point mutations and combined-site mutations of CV-B5/F using reverse genetics methods.

### Animals

Three-day-old BABL/c suckling mice, 4-week-old BALB/c nude mice, 4- to 6-weeks-old C57BL/6 mice, and 4- to 6-weeks-old BALB/c mice were supplied by the Laboratory Animal Center of the NIFDC. Animal research was conducted in compliance with the Animal Welfare Act and was approved was by the Ethics Committee of the NIFDC, China (approval no. 2022-B002). All the institutional and national guidelines for the care and use of laboratory animals were followed.

### Virus amplification, low-temperature passage, and titration

CV-B5/F was amplified and cultured in Vero cells (37°C, 5% CO_2_). When the CPE reached 90% or higher, the cells were transferred to correspondingly sized centrifuge tubes, subjected to freeze-thaw cycles three times, and centrifuged at 3,000 × *g* for 30 min at 4°C, and the viral supernatant was carefully collected as CV-B5/F. It was then aliquoted and stored at −80°C for later use. The amplified virus was cultured at 33°C in an atmosphere of 5% CO_2_, and the CV-B5/F strain was continuously cultured for 30 passages. The harvested viral supernatants were sequentially named as the 1st generation (F-P1) through to the 30th generation (F-P30). The viruses from each passage were stored at −80°C for later use. The harvested viruses were subjected to virus titer determination by cell culture infectious dose 50 (CCID_50_). In particular, the virus was diluted 10-fold using MEM maintenance medium (2% FBS), and each dilution was inoculated into pre-prepared Vero cells, which were then placed at 35°C in a 5% CO_2_ incubator. After seven days, the results were observed, the CPE was determined, and the virus titer was calculated using the Reed-Muench method.

### Suckling mice viral challenge to test viral virulence

The viral virulence was determined using 3-day-old BABL/c suckling mice, with 6–8 mice per group (including the mother mouse), using intraperitoneal inoculation with a volume of 60 μL per mice, and the negative control group was inoculated intraperitoneally with 60 μL virus dilution fluid. The mice were observed continuously for 21 days, with the weight of each litter measured every other day, and the survival and clinical symptoms of the suckling mice were observed and recorded daily. The clinical symptom scoring criteria were as follows: 0, health; 1, emaciation; 2, forelimb paralysis; 3, hindlimb paralysis; 4, quadriplegia; and 5, moribund or death.

### Viral genome sequencing

Total viral RNA was extracted from the viral harvest fluid from each strain using the MagMAX-96 viral isolation kit. Total RNA was then reverse transcribed to cDNA using the SuperScript III first-strand synthesis system for reverse-transcription PCR and random primers. cDNA was PCR amplified using the segmented amplification method. The first segment, approximately 3,300 bp in length, was amplified using CV-B5/F 1-F (5′-CAAGAATTGCGGCCGCGTAATACGACTCACTATAGGTTAAAACAGCCTGTGGGTTGTTCCCACC-3′) and CV-B5/F 1-R (5′-GCACCAGTGGTTTGCATGGTC-3′), and the second segment, approximately 4,200 bp in length, was amplified using CV-B5/F 2-F (5′-GACCATGCAAACCACTGGTGC-3′) and CV-B5/F 2-R (5′-AACATGAGAATTGTCGACTTTTTTTTTTTTTTTTTTTTTTTTTTTTTTTTTTTT-3′). The amplified segmented products were sent to Sangon Biotech for sequencing, using the two pairs of amplification primers mentioned earlier. The sequencing results were then compared.

### Construction of cDNA infectious clones

Using the CV-B5/F cDNA infectious clone constructed in our laboratory as a template,^31^ we designed two pairs of primers; the sequences containing mutation sites were synthesized by Sangon Biotech, to amplify two fragments containing mutation sites, with primers for each mutant strain, as shown in Table S3. The primer pairs pSVA-F (5′-CTCCGCATTCGGTGCGGAAAAAAAAAAAAAAAAAAAAAAAAAAAAAAAAAAAAAAGTCGACAATTCTCATGTT-3′) and pSVA-R (5′-GGTGGGAACAACCCACAGGCTGTTTTAACCTATAGTGAGTCGTATTACGCGGCCGCAATTCTTG-3′) were used for PCR amplification of the PSVA vector. The two CV-B5 target fragments obtained were mixed with a linearized PSVA vector at a molar ratio of 2:2:1 in 5× In-Fusion HD enzyme premix and placed in a PCR machine, at 50°C for 15 min, to perform homologous recombination. The transformation was performed according to the instructions for Trans109 Chemically Competent Cell. After transformation, the competent cells were centrifuged at 900 × *g* for 5 min, the supernatant was discarded, and the cells were resuspended in ∼200 μL of culture medium, evenly spread on agar plates with Amp resistance and incubated overnight in a 37°C incubator. The next day, colony growth was observed, and single clones were picked for bacterial liquid identification. The bacterial culture for PCR identification of specific single clones was incubated overnight at 37°C with shaking at 200 rpm/min. Plasmid extraction was performed according to the steps in the endotoxin-free plasmid mini-prep medium volume Kit manual. A nucleic acid protein analyzer (model: NanoDrop One) was used to detect DNA concentration. The plasmid was sequenced by Sangon Biotech. The sequencing primers used are listed in Table S4.

### Virus rescue

HEK-293T cells were seeded at a density of 1 × 10^6^ cells/well in a 6-well plate and incubated until they were more than 80% confluent. The jetPRIME kit transfection reagent was used to cotransfect the CV-B5/F plasmid (1.5 ng/μL) and T7 plasmid (1.5 ng/μL) into HEK-293T cells, and the cells were cultured at 37°C in a 5% CO_2_ incubator for 72 h. The cell suspension was subjected to freeze-thaw cycles three times and centrifuged at 3,000 × *g* for 30 min at 4°C; 100 μL of the supernatant obtained was used to inoculate a monolayer of Vero cells, and CPE was observed. After the typical CPE was evident, the cell suspension was collected, subjected to freeze-thaw cycles three times, and centrifuged at 3,000 × *g* for 30 min at 4°C, and the supernatant was collected as the viral harvest fluid. The viral harvest fluid from successful transfection was passaged for five generations in Vero cells to obtain a stable mutant strain. The stably passaged strains were used for determination of the viral titer (as described previously). Each mutant strain, thus obtained, was amplified in segments, sequenced, and analyzed. After verification, the strains were aliquoted and stored in a −80°C freezer.

### Indirect immunofluorescence assay

Vero cells were infected with the virus at an MOI of 0.01 for approximately 12 h. The culture medium was aspirated; the cells were fixed with 4% formaldehyde, blocked, and incubated with CV-B5 mouse monoclonal antibody, followed by incubation with an Alexa Fluor 488-labeled anti-mouse antibody. Fluorescence emission was observed under a confocal microscope.

### Western blot analysis

Vero cells were infected with the virus at an MOI of 0.01 for approximately 24 h. The cells were pelleted, resuspended, subjected to three freeze-thaw cycles, and centrifuged at 3,000 × *g* for 30 min. The viral supernatant was collected. The protein was analyzed using western blotting. Mouse monoclonal antibodies against the structural proteins VP0+VP2 and VP1 of CV-B5 were used as the primary antibodies, and a monoclonal antibody against mouse immunoglobulin G was used as the secondary antibody.

### qPCR detection of viral load in suckling mice

Three-day-old BALB/c suckling mice were infected with CV-B5/F and the attenuated VP1-N157K strain at the same dose of 1.896 × 10^7^ CCID_50_ (60 μL per mouse). Under sterile conditions, the pancreas, cerebrum, spine, and hind legs were collected at 6, 24, 48, 72, 120, and 168 h. Tissues were homogenized using an OMNI Bead Ruptor 24 Elite multifunctional biological sample homogenizer. After homogenization, the samples were centrifuged at 12,000 × *g* for 15 min, and the supernatant was collected. Total viral RNA was extracted from the supernatant using the MagMAX-96 viral solation kit. qPCR experiments were performed using the TB Green Premix Ex Taq II kit, with CV-B5/F as the template, with primers designed based on the conserved sequence of its 5′-untranslated region for dye-based qPCR detection. The primers sequences were as follows: qPCR-F: 5-′GTCCGTGTTTCTTTTAATTTTATACTGGCTGC-3′; qPCR-R: 5′-GTTGGATACCGGATGGCCAATCC-3′.

### Preparation of RNA standard curve

The cDNA clone of CV-B5/F was amplified using CV-B5/F 1-F and CV-B5/F 2-R to obtain full-length CV-B5/F (approximately 7.400 bp). RNA was obtained as a standard using *in vitro* transcription (T7 RiboMAX Express large-scale RNA production system). A 10-fold serial dilution of the original RNA solution was performed, and 10^5^–10^9^ dilutions were selected as standard curve points. The copy number of RNA standards was calculated as follows: number of copies (copies/μL) = (6.02 × 10^23^ copies/mol) × (RNA ng/μL×10^9^)/(Length (bp) × 340 Da/bp). The Ct values obtained for each sample were substituted into the standard curve to calculate the viral load in the tissue.

### Tissue pathology

CV-B5/F and the attenuated VP1-N157K strain were used to infect 3-day-old BALB/c suckling mice at the same dose (1.896 × 10^7^ CCID, 50/mL), 60 μL per mice. When the suckling mice in the CV-B5/F infection group were in a moribund state (approximately the 5th day after infection), three mice from each group at the same time point were taken for pathological studies, and three blank control group suckling mice were also set up. Hematoxylin and eosin staining was performed on the pancreas, brain, spine, hind legs, and other tissues and organs, under sterile conditions.

### Cell entry bypass assay

293T cells were seeded at a density of 1 × 10^6^ cells/well in a 6-well plate and cultured overnight at 37°C until they were approximately 80% confluent. Five microgram mRNA from the CV-B5/F or VP1-N157K strain was transfected into the cells using Lipofectamine RNAiMAX transfection reagent, and the cells were washed twice after 3 h. The supernatant was collected, and the cell pellet was washed three times with PBS after 8 h, resuspended in 200 μL PBS, and subjected to freeze-thaw cycles three times to release the virus. The Reed-Muench method was used to detect the viral titer inside and outside the package. The cell pellet was collected at 24 h, and western blotting was used to detect the expression of viral proteins.

### Pull-down assay for virus and receptor binding

ProFound pull-down Poly-His protein: protein interaction kit (catalog number: 21277) was purchased from Thermo Scientific. After equilibrating HisPur cobalt resin, CAR protein was added to His tag or DAF protein with Tag and incubated on a rotating platform for 30 min at 4°C to immobilize on HisPur cobalt resin. The HisPur cobalt resin with bait protein fixed on it was washed five times; then, purified CV-B5/F or VP1-N157K strain was added, and the mixture was incubated on a rotating platform for more than 1 h at 4°C. The HisPur cobalt resin was washed five times with the captured protein, and the interacting proteins were eluted using imidazole elution buffer. The protein content was further analyzed using gel electrophoresis.

### SPR experiment

All SPR experiments were conducted in Phosphate buffered saline with tween-20 (pH 7.4) at 25°C using a BIAcore T200 instrument at a flow rate of 30 μL/min. The affinities of the CV-B5/F and VP1-N157K strains for the DAF receptor protein were measured separately with recombinant DAF protein immobilized on a CM5 chip at a surface density of 100 resonance units. CV-B5/F and VP1-N157K were then flown over different channels. The binding kinetics were analyzed using the BIA evaluation software, version 3.0, employing a 1:1 binding model.

### Assay of virus attachment on cell surface

Vero cells were seeded in a 6-well plate at a density of 1 × 10^6^ cells/well and cultured overnight at 37°C. The wild-type CV-B5/F and the attenuated VP1-N157K strains were used to infect Vero cells at MOIs of 1, 0.1, and 0.01, and the cells were incubated at 4°C for 3 h. The supernatant was discarded, and the cells were washed three times with PBS, resuspended in 400 μL PBS, and subjected to freeze-thaw cycles three times. Viral RNA was extracted, and the viral copy number in the supernatant was detected.

### Cytotoxicity assay

CT26.WT, CT26.WT-CAR, LLC, LLC-CAR, and other cells (100 μL/well) were plated in 96-well plates, at a density of 1 × 10^4^ cells/well (excluding the edge wells), and cultured at 37°C in a 5% CO_2_ incubator. The viral titers of CV-B5/F and the attenuated VP1-N157K strain were adjusted to the same level (MOI = 100). A 10-fold serial dilution was performed with a total of 7 dilutions. Once the cell density in the 96-well plate reached 70%–80%, diluted virus was slowly added to the corresponding wells (100 μL/well) in triplicate and cultured at 37°C in a 5% CO_2_ incubator; three negative control wells (with cells, with maintenance medium) and three blank controls (without cells, with maintenance medium) were also set. The cells were incubated for 48 h, the Cell Counting Kit-8 (CCK-8) reagent (10 μL/well) was added to the wells, and, when the OD_450_ value of the negative control wells reached approximately 1.0, the absorbance of each well was measured at 450 nm. The cell survival rate was calculated using the following formula:

Cellsurvivalrate(%)=(ExperimentalwellODvalue−BlankwellODvalue)/(ControlwellODvalue−BlankwellODvalue).

### *In vivo* oncolysis experiment

H1299 cells were expanded *in vitro* and subcutaneously implanted into 4- to 6-week-old female BALB/c nude mice. CT26.WT and CT26.WT-CAR cells were also expanded *in vitro* and subcutaneously implanted into 4- to 6-week-old female BALB/c mice. LLC and LLC-CAR cells were expanded *in vitro* and subcutaneously implanted in 4- to 6-week-old female C57BL/6 mice. After approximately 10 days, the tumor diameter reached approximately 5–6 mm, indicating successful implantation. The CV-B5/F and attenuated VP1-N157K strains were intratumorally injected at a titer of 2.14 × 10^7^ CCID_50_/mL (200 μL per mouse), with at least 5 mice per group. The strains were injected every other day for 5 consecutive times. The negative control group was injected with 200 μL per mouse of culture medium maintenance solution intratumorally. Before injecting, the long and short diameters of the tumor were measured to calculate the tumor size using the following formula:

Tumorvolume(cubicmillimeters)=(longdiameter(mm)×shortdiameter(mm)×shortdiameter(mm))/2.

### Data analysis

Data from this experiment were statistically analyzed using GraphPad Prism version 9. One-way ANOVA variance was used to assess the statistical differences between groups, and multiple comparisons were conducted using the Tukey method to determine the significance of differences between pairs of groups. A significance level of *p* < 0.05 was used to reject the null hypothesis that there was no difference between the groups tested.

## Data availability

All research data supporting the findings of this study are available upon reasonable request by readers.

## Acknowledgments

We thank Meirong Gu, Yinlong Liu, and Wei Jian in Minhai Biotechnology Co. Ltd (Beijing, China) for providing CV-B5/F and VP1-N157K purified viruses and Ge Wang, Chunying Dai, and Pengyun Hou in Autobio Diagnostics Co. Ltd (Zhengzhou, China) for providing mouse monoclonal antibodies against CV-B5. This work was supported by the CAMS Innovation Fund for Medical Sciences (2021-I2M-5-005).

## Author contributions

L.S., Q.M., Z.L., and Y.Y. designed the studies and wrote the manuscript. L.S. and B.C. performed the experiments, analyzed data, and drew the picture. K.L. performed part of the construction of cDNA infectious clones. X.W. conducted part of the western blotting. Q.G. and C.H. conducted part of the suckling mice viral challenge to test viral virulence. F.G., X.H., C.H., J.Z., Y.L., G.L., Y.F., Y.W., and Q.W. assisted with animal experiments. L.S., B.C., and Q.W. involved in manuscript preparation. All authors have read and approved the article.

## Declaration of interests

The authors declare no competing interests.

## Supplemental Information

Document S1. Figures S1–S4 and Tables S1–S4

Document S2. Article plus supplementary information
