# Supplementary material for: An attenuated coxsackievirus B5 mutant carrying VP1-N157K retains oncolytic potency against non-small cell lung cancer
Source: Mol Ther Oncol. 2025 May 23;33(3):200999. doi: 10.1016/j.omton.2025.200999 (PMC12861664; doi:10.1016/j.omton.2025.200999)
Supplement: Document S1. Figures S1–S4 and Tables S1–S4 [file mmc1.pdf]

OMTON, Volume *33*

Supplemental information

An attenuated coxsackievirus B5 mutant carrying VP1-N157K retains oncolytic potency against non-small cell lung cancer

Lifang Song, Bopei Cui, Qiushuang Gao, Chaoying Hu, Qian wang, Jialu Zhang, Yajing Li, Guanxing Liu, Yulong Fu, Ying Wang, Kelei Li, Xiaotian Hao, Fan Gao, Xing Wu, Qunying Mao, Zhenglun Liang, and Yongxin Yu

Supplemental Material

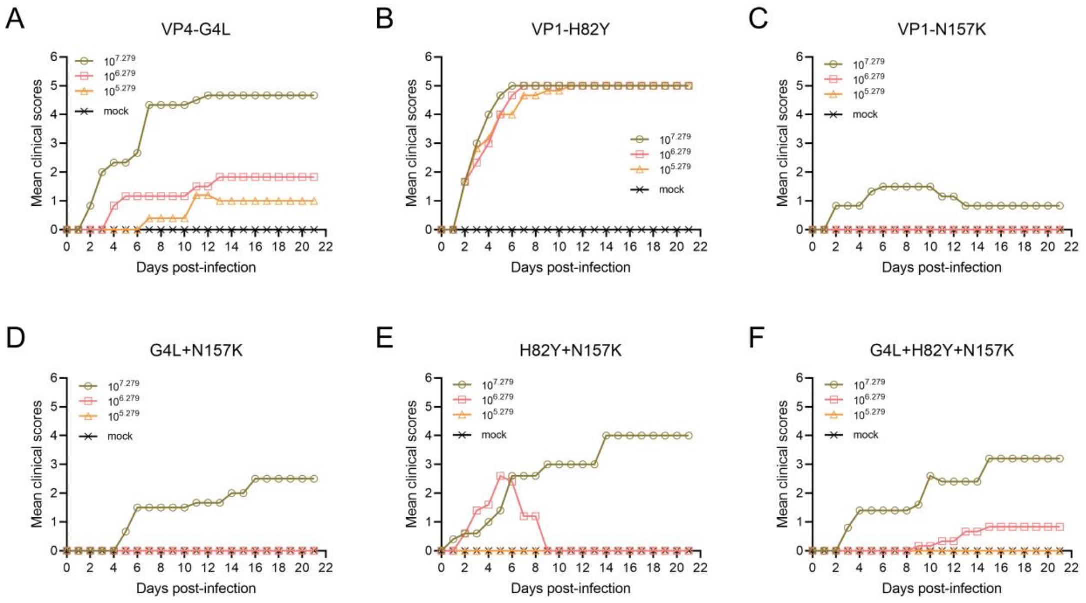

Figure S1. Mean clinical scores of suckling mice for mutants

(A-F) Mean clinical scores of 3-day-old BABL/c suckling mice challenged with the single-site mutant strains VP4-G4L (A), VP1-H82Y (B), VP1-N157K (C), and the combined-site mutant strains G4L+N157K (D), H82Y+N157K (E), and G4L+ H82Y+N157K (F), at doses of $10^{7.279}$ $CCID_{50}$/mL (high dose), $10^{6.279}$ CCID50/mL (medium dose), and $10^{5.279}$ $CCID_{50}$/mL (low dose), observed for 21 days (n=6).

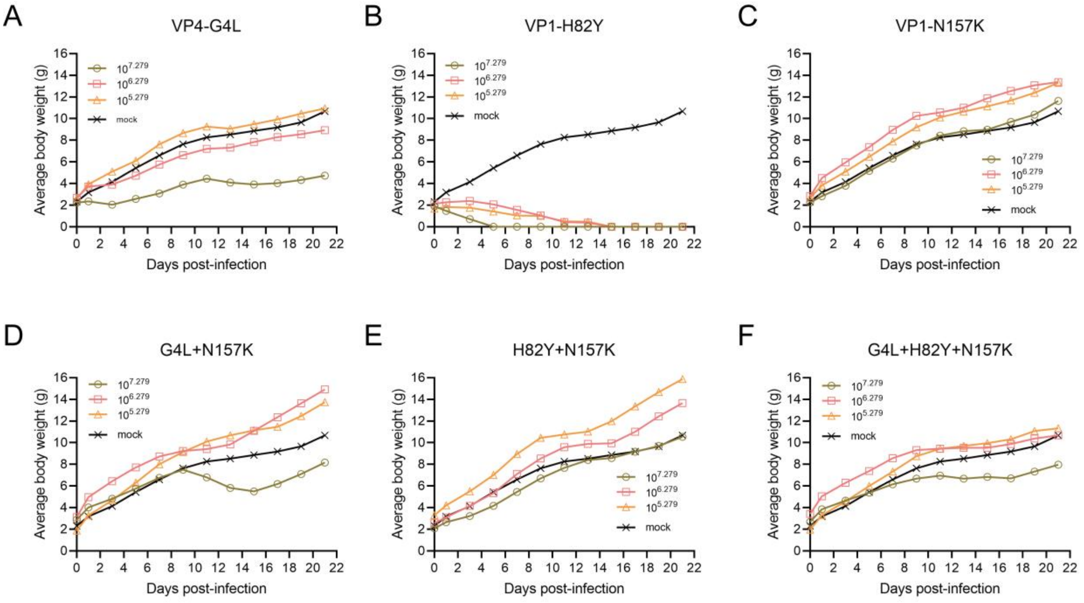

Figure S2. Average body weight of suckling mice for mutants

(A-F) Average body weight of 3-day-old BABL/c suckling mice challenged with the single-site mutant strains VP4-G4L (A), VP1-H82Y (B), VP1-N157K (C), and the combined-site mutant strains G4L+N157K (D), H82Y+N157K (E), and G4L+ H82Y+N157K (F), at doses of $10^{7.279}$ $CCID_{50}$/mL (high dose), $10^{6.279}$ CCID50/mL (medium dose), and $10^{5.279}$ $CCID_{50}$/mL (low dose), observed for 21 days (n=6).

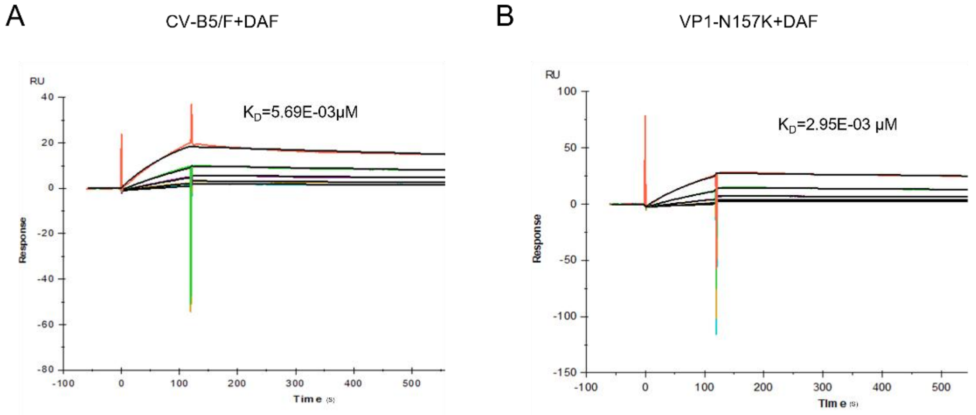

Figure S3. The binding capacity of the VP1-N157K and CV-B5/F strains to DAF receptors, respectively.

(A) The binding capacity of the wild-type strain CV-B5/F to the DAF receptor;(B) The binding capacity of the attenuated strain CV-B5/VP1-N157K to the DAF receptor.

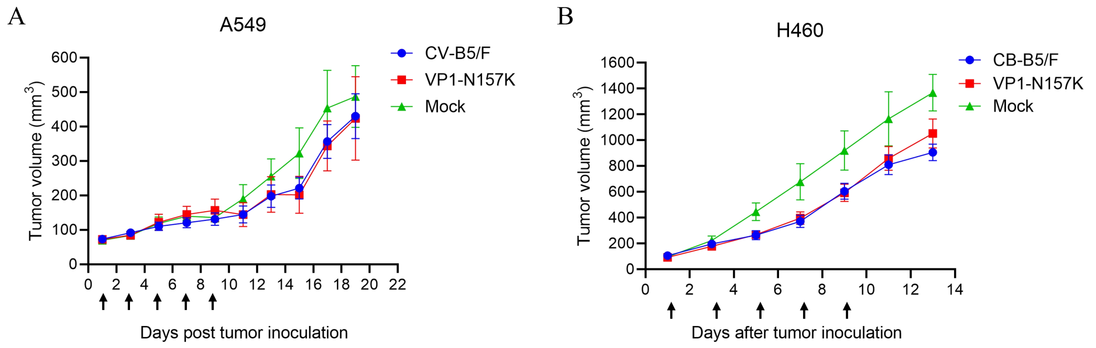

Figure S4. Oncolytic activity of the VP1-N157K attenuated strain in A549 and H460 lung cancer mouse models.

(A-B) The oncolytic efficacy in vivo of the A549 tumor model (A) and H460 tumor model (B) following five consecutive intratumoral injections of the CV-B5/F strain and the VP1-N157K attenuated strain at a dose of 5.12×10^6 $CCID_{50}$/mL.

Table S1 The virus titers of low-temperature passaging

Viruses	lg$CCID_{50}$/mL
CV-B5/F	8.33
F-P10	8.75
F-P20	8.50
F-P30	8.33

Table S2 Viral titers of mutant strains

Viruses	lg$CCID_{50}$/mL
VP4-G4L	8.59
VP1-H82Y	8.67
VP1-N157K	8.50
G4L+ N157K	8.59
H82Y+ N157K	8.71
G4L+ H82Y+ N157K	8.67

Table S3 PCR primers for each mutant strain

Mutant Strain	Primer	Sequence (5′-3′)	Position	Length
VP1-G4L	CV-B5 1-F	CAAGAATTGCGGCCGCGTAATACGACT<br>CACTATAGGTTAAAACAGCCTGTGGGT<br>TGTTCCCACC	1	802
	4-R	CTGGGTCGACACTAGAGCTCCCATT	802	
	4-F	ACACAGAAAAATGGGAG<br>CTCTAGTGTCG	768	6724
	CV-B5 2-R	AACATGAGAATTGTCGACTTTTTTTTTT<br>TTTTTTTTTTTTTTTTTTTTTTTT	7492	
VP1-H82Y	CV-B5 1-F	CAAGAATTGCGGCCGCGTAATACGACT<br>CACTATAGGTTAAAACAGCCTGTGGGT<br>TGTTCCCACC	1	2379
	82-R	CCGTCAGTGCCATAATTTTTGTATGTGG<br>TGTA	2739	
	82-F	TACACCACATACAAAAATTATGGCACT<br>GACG	2708	4784
	CV-B5 2-R	AACATGAGAATTGTCGACTTTTTTTTTT<br>TTTTTTTTTTTTTTTTTTTTTTTT	7492	
VP1-N157K	CV-B5 1-F	CAAGAATTGCGGCCGCGTAATACGACT<br>CACTATAGGTTAAAACAGCCTGTGGGT<br>TGTTCCCACC	1	2967
	157-R	TGCCAACTGTAGCTTTTTACTTTTGTGG<br>GCA	2967	
	157-F	TGCCCACAAAAGTAAAAAGCTACAGTT<br>GGCA	2937	4525
	CV-B5 2-R	AACATGAGAATTGTCGACTTTTTTTTTT<br>TTTTTTTTTTTTTTTTTTTTTTTT	7492	

Table S4 Sequencing primers for the CV-B5/F strain

Primer	Sequence (5′-3′)	Position	Length
F1	TTAAAACAGCCTGTGGGTTGTT	1	950
R1	GTTTAGTGCAGGCATGGATT	949	
F2	GACCCGGGGAAATTCACGGA	887	1560
R2	TTGAAAGAAGCTGTCCTGTTT	2482	
F3	GTACCAAACTAACATCGTGGT	2310	1006
R3	GGATTGCTGTCCAAAGGCACC	3317	
F4	TGCCAGTACCAGAAAGC	3209	1842
R4	CTCCCTGTACACTGGAGGACC	5050	
F5	TACAACCACAGGCATAGCGTA	4892	958
R5	TCGAGTTCTCAATGAACTCAATC	5940	
F6	GTGGGAATGGTCATCAAGG	5849	1555
R6	TTTTCCGCACCGAATGCGGAGAAT	7404	
